# Integrated mechanical environment of pre- and post-rupture fault and asperity origin of the 2011 giant Tohoku-Oki earthquake

**DOI:** 10.1038/s41598-022-25433-6

**Published:** 2022-12-08

**Authors:** Zhoumin Xie, Yongen Cai

**Affiliations:** 1 National Institute of Natural Hazards, MEMC, Beijing, China; 2 Institute of Theoretical and Applied Geophysics, School of Earth and Space Sciences, Peking University, Beijing, 100871 China

**Keywords:** Natural hazards, Solid Earth sciences

## Abstract

It is a key to know mechanical environment (ME) of pre- and post-rupture fault of giant earthquakes at subduction zones for predicting earthquake and tsunami disaster. However, we know little about its details till now. In this paper, using the inverted stress change three hours before and three hours after the mainshock in the rupture zone of the 2011 Tohoku-Oki *M*_w_ 9.0 earthquake, we show a quantitative integrated ME in the rupture zone, including principal stress, pore-fluid pressure and friction strength. We discover from this environment a large asperity composed of two asperities induced by relatively high friction coefficients and relatively lower pore-fluid pressures. The integrate ME quantitatively explained the reasons of the overshoot and relatively lower shear strength of the trench, which caused huge displacement and tsunami at the trench. We suggest that the asperities favor the horst and graben structure system which provides a geology environment for interseismic stress accumulation and thus for breeding the megathrust tsunami earthquake.

## Introduction

Up to now all recorded earthquakes with magnitude of greater than or equal to *M*_w_ 9.0 occurred on megathrust faults at subduction zones^1–4^. We know little about the mechanical environment (ME) of faults to breed them in quantitative details, such as the magnitude and distribution of stress, pore-fluid pressure and friction strength. The *M*_w_ 9.0 Tohoku-Oki earthquake of March 11, 2011 provided an opportunity to probe this issue. We have known better about the rupture process and slip distribution of the earthquake by using the dense global positioning system (GPS) networks^5^ and the seafloor displacement data close to the Japan trench^6–8^. The surprisingly large slip up to 60 m on the shallow portion of the fault has been qualitatively explained with dynamic overshoot^9^ or low frictional resistance of the fault^10–12^. However, the integrated ME to breed the earthquake is rarely reported to quantitative details.

After the mainshock, for further understanding the giant earthquake mechanism, the Japan Trench Fast Drilling Project (JFAST, Integrated Ocean Drilling Project Expedition 343 and 343 T) drilled to the ruptured fault at shallow depth and made borehole measurements. The borehole breakouts revealed that the azimuth of local horizontal maximum stress (intermediate principal stress) is in the direction of N139° ± 23°^10^, which is consistent with the plate convergence direction^13^ and its magnitude is about 87 MPa estimated by assuming Anderson's stress state and a vertical stress calculated from a sediment density profile as maximum principal stress^10^. The rock friction experiments were conducted on samples retrieved from the borehole at the depth close to the plate-boundary^11^, the shear stress obtained from the experiments is 1.32 and 0.22 MPa, respectively, for the in-situ condition under permeable and impermeable cases, which correspond to values for the in-situ apparent coefficient of friction of 0.19 and 0.03. The contribution to pore-fluid pressure on the fault is little known, it is considered as nearly hydrostatic pore pressure at a shallow part and nearly lithostatic pressure at a deep part of the plate interface based on a numerical simulation^14^.

These results above give us some insight into the ME of the Tohoku-Oki earthquake though only at some locations on the fault. However, the details of the ME in pre- and post-rupture fault of the earthquake are still unclear, which is very important for understanding earthquake preparation, occurrence and earthquake prediction.

In consideration of the effect of pore-fluid pressure on fault stress state, the total static stress change can be taken as the sum of changes in the total effective stress and in the pore pressure^15^, which may allow us to recover ME of earthquake fault (see “Methods”) if it is assumed that rock ruptures to obey the Coulomb failure criteria^16^ and that the fault is in an undrained state during earthquake rupture^17^.

**Figure 1:**
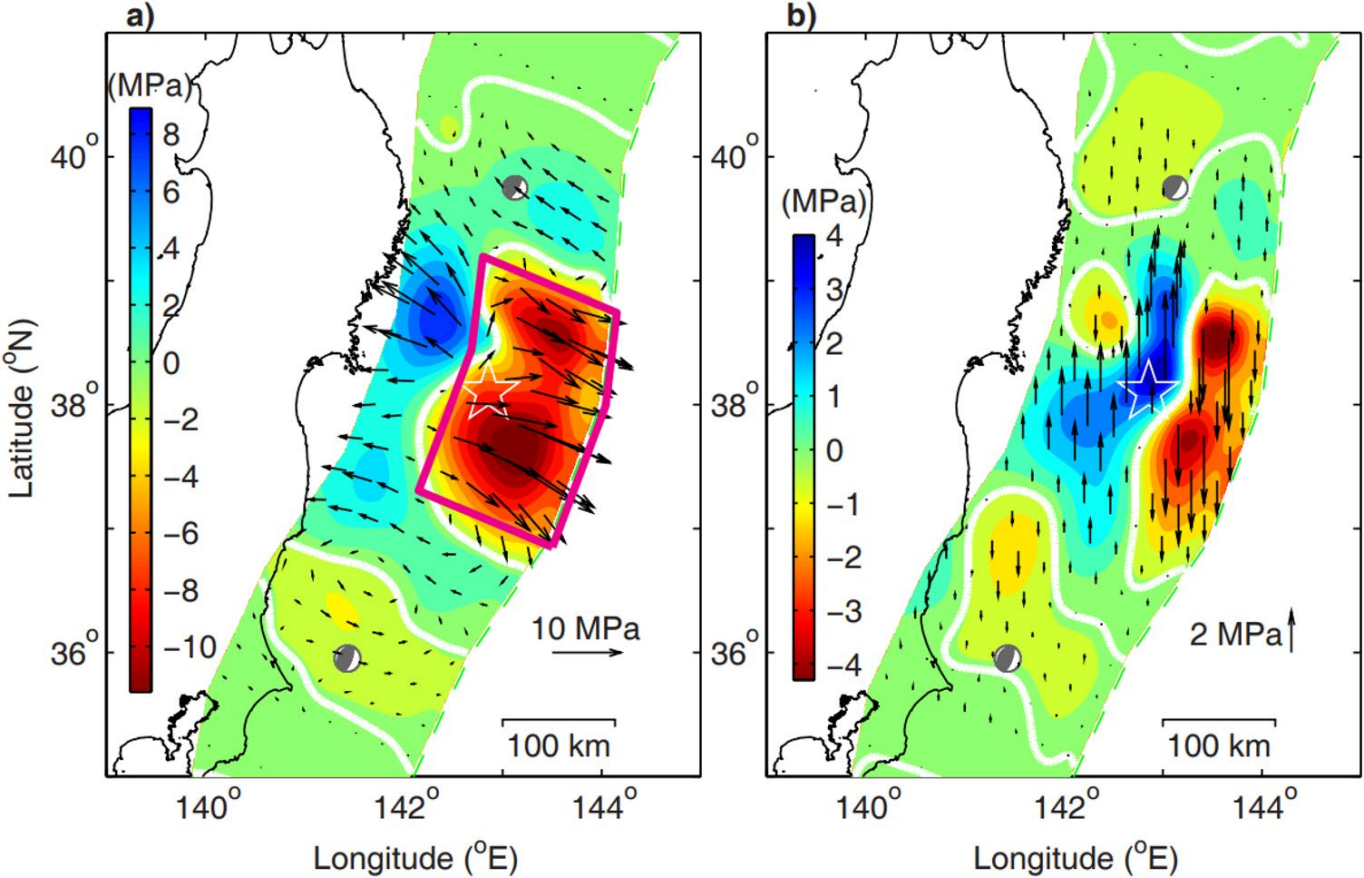
Inverted static coseismic stress changes of the Tohoku-Oki earthquake. (**a**) and (**b**) are the shear and normal stress changes of the Tohoku-Oki earthquake along the fault^18^, respectively, the stress change surrounded by red line are used to recover the ME of the earthquake. The white zero-shear stress drop contour encompasses the rupture areas of the mainshock and the largest M_w_ 7.8 aftershock. Beach balls in the north-east and south-west show focal mechanisms of the aftershocks with M_w_ 7.4 and M_w_ 7.8 occurred at UTC 06:08 and 06:15 on 11 Mach, respectively. The star indicates the hypocenter of the mainshock, the arrows denote the magnitudes and directions of the inversed stresses.

In this study, our purpose is to recover the integrated ME of the pre- and post-rupture fault of the Tohoku-Oki earthquake using the static fault stress change inverted by the coseismic deformation 3 h before and 3 h after the earthquake^18^. The region enclosed by the contour of zero-shear stress drop in Fig. 1a is the fault rupture zone of the Tohoku-Oki earthquake, in which the integrate ME is to be recovered in the study. The method to recover the ME is described in “Methods” section.

## Results

Using the coseismic stress change (Fig. 1a,b) of the fault rupture zone of the Tohoku-Oki earthquake as loading of the boundary value problem^18^, we solved the stress tensor change (Table S1) at the fault firstly, and then using the stress tensor change and the method proposed in the study, we recovered the integrated ME (Table S2) in the rupture zone three hours before and three hours after the mainshock. The integrated ME includes effective principal stress state (magnitude and direction), internal frictional coefficient and coseismic pore-fluid pressure change which indirectly determined by normal stress changes and Skempton coefficient, a parameter to reflect a capacity of pore-fluid pressure change under one unit change of total mean normal stresses. This parameter is related to rock compressibility, the larger the rock compressibility is, the larger the Skempton coefficient^17,19^.

Considering that the cumulated stress almost all released after the mainshock^8^, we take the lithostatic pressure as the total vertical stresses and use the recovered pore-fluid pressure change and the total vertical effective stress, we obtained the pre- and post-seismic pore-fluid pressures (Table S3). The total principal stresses (Table S3) in the fault rupture zone before and after the Tohoku-Oki earthquake are calculated by using the pre- and post-seismic pore-fluid pressures and the recovered pre- and post-seismic principal effective stresses.

**Figure 2:**
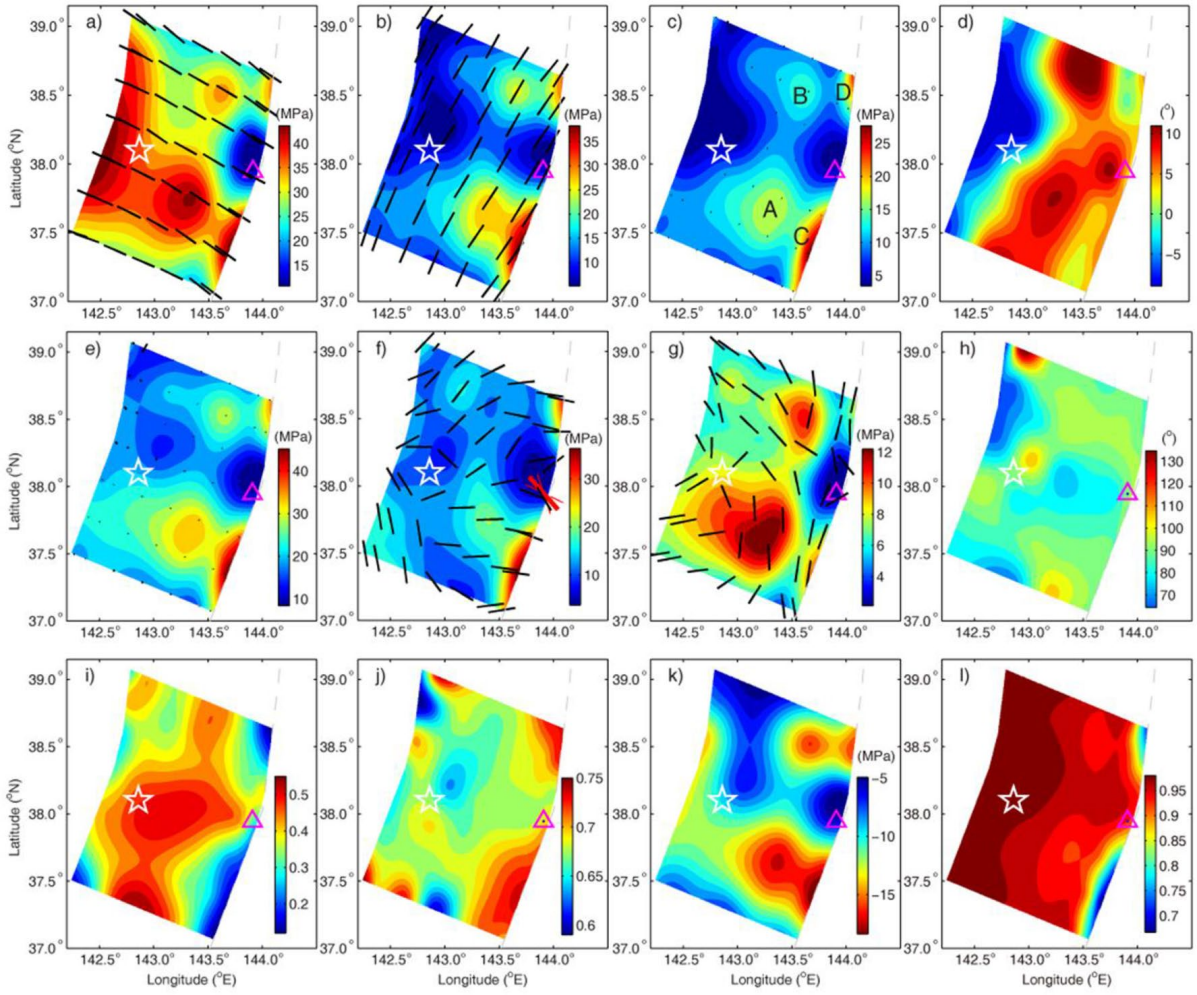
Recovered ME in the fault rupture zone of the Tohoku-Oki earthquake. (**a**–**d**) Maximum (*S*_1_), intermediate(*S*_2_), minimum(*S*_3_) principal effective stresses and the dip angle of *S*_1_ before the mainshock, respectively. (**e**–**h**) *S*_1_, *S*_2_, *S*_3_, and the dip angle of *S*_1_ after the mainshock, respectively. (**i**) Friction coefficient. (**j**) Skempton coefficient, a parameter to reflect a capacity of pore-fluid pressure change under one unit change of mean total compressive stress. (**k**) Pore-fluid pressure change. (**l**), Pore-fluid pressure ratio of the pre-fault rupture. Star denotes the hypocenter of the mainshock. Black bars, normalized principal stresses viewed from above, full length indicates horizontal, and length decreases with increasing the dip angle. A thick and two thin red bars in (**f**) represent the predicted maximum horizontal stress direction range from the borehole breakout and error angles. Triangle denotes the location of IODP JFAST site.

The integrated ME recovered is shown in Fig. 2. Before the mainshock, the principal effective stresses (positive for compressure) all show the four regions of abnormal stress in the up-dip portion from the hypocenter, referred to as A, B, C and D marked in Fig. 2c for convenience. The maximum value of the recovered maximum principal effective stress (*S*_1_) with standard deviation (SD) is 42.7 ± 5.8 MPa in the Region A at the depth of 14.5 km below sea level. The direction of *S*_1_ is N124.1° ± 8.4° (Fig. 2a), consistent with the borehole breakouts average^13^ and the direction of the Pacific plate convergence, and its dip angle is 9.7° ± 2.6° (Fig. 2d), close to the horizontal direction. The directions of the recovered intermediate principal effective stress (*S*_2_) and the minimum principal effective stresses (*S*_3_) are nearly along the trench and vertical (Fig. 2b,c), respectively. This principal stress state is consistent with sign of the condition required for a thrust earthquake. The values of *S*_1_, *S*_2_ and *S*_3_ on average in the fault rupture zone are 32.4 ± 3.5, 19.7 ± 4.0, 10.5 ± 4.3 MPa, respectively, which are about one order of magnitude smaller than the total principal stresses (Table S3).

After the mainshock, the four regions of the abnormal stress still exist for *S*_1_ and *S*_3_ (Fig. 2e,g), but the region D disappears for *S*_2_ in Fig. 2f. The average direction of *S*_1_ becomes from nearly horizontal before the mainshock to nearly vertical. The average direction of *S*_3_, from vertical to nearly along the trench and that of *S*_2_ from along the trench to nearly normal to the trench. The values of *S*_1_, *S*_2_ and *S*_3_ on average are 22.4 ± 4.4, 16.0 ± 4.5, 6.4 ± 1.3 MPa, respectively. The principal effective stresses close to the vertical direction (Fig. 2h) and nearly normal to the trench increased by 12 MPa and decreased by 16 MPa, respectively, after the earthquake.

The total intermediate principal stress at site C0019 of the JFAST, marked by a triangle in Fig. 2f, is 88.9 MPa (Table S3) at the depth of 7.5 km, which is close to 87 MPa estimated by the JFAST based on assuming Anderson's stress state and a vertical stress calculated from a sediment density profile as maximum principal stress^10^. Its orientation is about N149.1° ± 13°E (marked by a black bar in Fig. 2f), close to the range N139°E ± 23° of the maximum horizontal stress (marked by a thick pink bar and two fine pink bars in Fig. 2f)^10^, which is determined by the borehole breakout at the JFAST site C0019.

The shear stress along the fault dip is 1.1 MPa (Table S4) at 7.5 km below the triangle sign, which is determined by the recovered effected principal stresses. This value is consistent with that of 1.32 MPa from the rock friction experiments on samples retrieved from the borehole at the depth close to the plate-boundary^11^ for the in-situ condition under permeable case.

The recovered frictional coefficient (Fig. 2i) shows an irregular belt of maximum value of ~ 0.55, which hosts the region of the maximal shear stress drop^18^. The lower value of the frictional coefficient of less than 0.3 is in the regions C and D, close to the trench. The distribution of Skempton coefficient (Fig. 2j) in the up-dip portion from the hypocenter is roughly consistent with that of the stress abnormal regions except that of the region B. Its larger value is in the regions C and D. Both the pore-fluid pressure change (Fig. 2k, Table S3) and the pore-fluid pressure ratio (Fig. 2l, Table S3), defined by pore-fluid pressure to lithostatic pressure, show four abnormally low areas corresponding to four abnormally high areas of the principal effective stresses. The pore-fluid pressure drop is 17.6 ± 3.2 MPa in the region C close to the trench at the depth of 7 km from the sea level and 16.1 ± 3.3 MPa at the depth of 14.5 km in the region A, respectively, which is close to the hypocenter of the earthquake. The average value of the pore-fluid pressure ratio before the fault rupture is 0.950, which is close to 0.965 estimated from force balance in a two-dimension model of Japan^20^.

## Discussion

The average difference between the shear stresses predicted by the recovered principal effective stresses and those inversed directly^18^ in the rupture area is less than 0.1 MPa (Table S4), and the stress accuracy loss caused by using plane sub-fault to replace curved sub-fault with maximum curvature of 0.005 is less than 1%. These indicate the integrated ME recovered is acceptable. The recovered ME is based on such a structure setting in which the fault geometry shape and rock properties have been determined and widely accepted. The former is consistent with the actual subduction zone as shown in Fig. S1a, and the latter is from PREM^18^. Theoretically, different structure setting will lead to different mechanical environments. Due to the availability of seismic tomography and the shape of the subduction zone revealed by earthquake distribution, the uncertainty introduced by the fault geometry shape we used is low. The uncertainty of rock properties is less than 1 MPa for the 6% P-wave velocity change in the stress model. These uncertainties allow us conclude that the integrated ME recovered is reliable.

**Figure 3:**
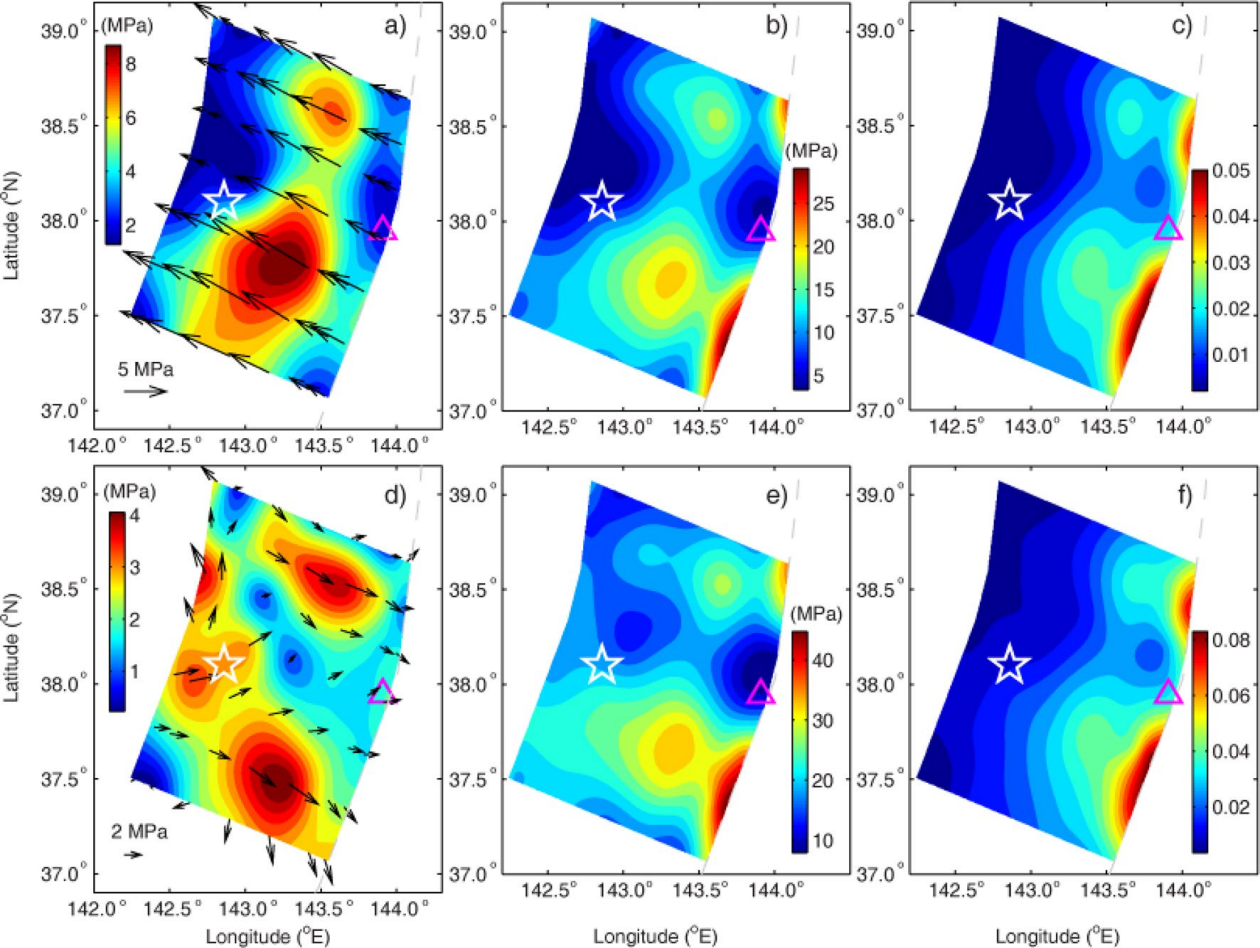
Predicted the shear and normal stresses and effective coefficient from the ME. (**a**,**b**) Pre-seismic shear and normal effective stresses along the fault, respectively. (**d**,**e**) Post-seismic shear and normal effective stresses along the fault, respectively. (**c**,**f**) Effective frictional coefficient of the pre-and post-fault rupture along the fault, respectively.

**Figure 4:**
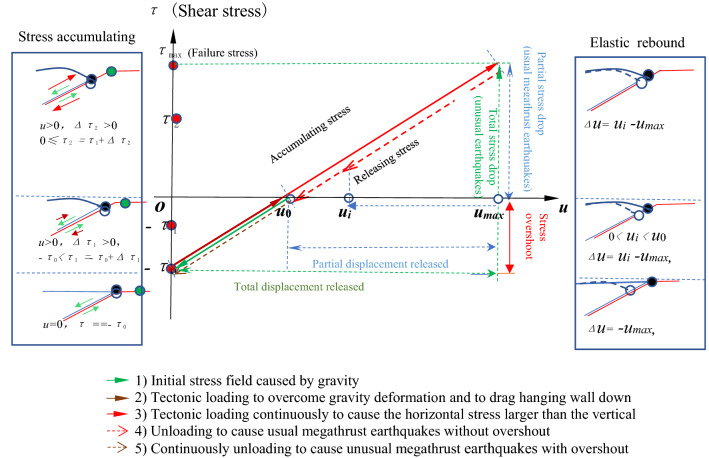
Accumulating and releasing progress of stress at a point on the fault surface of the hanging wall of the Tohoku-Oki earthquake.

The shear and normal stresses and the effective frictional coefficients on the pre- and post-rupture fault are shown in Fig. 3 (see details in Table S4), which are determined by the recovered pre- and post-seismic principal effective stresses. The shear stress represents shear strength of the fault because it meets Coulomb friction criteria^16^. The two maximum shear stresses in Fig. 3a along the fault dip direction before the mainshock are 8.4 and 7.1 MPa at the depth of 14.5 km in the regions A and B, respectively, which are less than the shear stress drops of 11.7 and 10.1 MPa in the same regions of the Tohoku-Oki earthquake^18^. The average shear stresses are 4.5 and − 1.7 MPa (Fig. 3a,d and Table S4) along the fault dip before and after the mainshock, respectively, the minus sign indicates that the direction of shear is opposite to the direction of the Pacific plate subduction. The difference of them is right the mean shear stress drop of 6.2 MPa inverted (Fig. 1a) in the rupture area, and the latter is almost equal to that the mean shear stress of 2.2 MPa in resisting hanging wall sliding caused by effective gravity before the earthquake (Table S3). This result quantitatively proves that there exists shear stress overshoot in the rupture area on the fault of the earthquake, i.e. the mainshock not only totally released the pre-seismic shear stress (4.5 MPa) to make thrusting deformation, but also released that (1.7 MPa) to overcome the deformation from gravity (Fig. 4). The former is consistent with the usual shear stress drop of large earthquakes in average^21^ on the plate boundaries. This result may explain why the mean total stress drop of 6.2 MPa inverted (Fig. 1a) is unusual (Fig. 4). It is the unusual shear stress drop that leads to surprisingly large slip on the shallow portion of the megathrust fault. The shear strength in the regions C and D at the trench is much lower than that in the A and B (Fig. 3a), which may be one of reasons why the rupture can reach to the trench and cause huge displacements there and huge tsunami.

From the pore-fluid pressure ratio (Fig. 2l) we find that there exist four lower abnormal regions before the mainshock, which are consistent to the high abnormal effective stress regions. It may be caused by the pore-fluid pressure in these regions, which is lower than that in their surroundings at the same depths (see Table S3). The regions with lower pore-fluid pressure may be explained by that there are more rock microcracks than those of their surroundings. The microcracks may be attributed to the rock dilatancy before the fault rupture^16^. It is the abnormal microcracks that lead to permissibility increase of these regions, which induces the lower pore-fluid pressure and the higher effective stress there. This explanation is supported by the higher value of Skempton coefficient *B* in the Fig. 2j, because higher *B* means higher rock compressibility^17,19^, and the higher the rock compressibility is, the more the rock microcreaks.

The effective frictional coefficient of pre-rupture fault (Fig. 3c) in regions A, B, C and D is higher than that in their surroundings. Its average of 0.016 is less than 0.029 after the mainshock (Fig. 3f, Table S3), the latter is consistent with 0.025 obtained from modeling heat flow data at the Japan Trench^22^. This may be explained by the relatively large pore-fluid pressure drop (Fig. 2k) after the mainshock.

High-resolution seismic lines crossing the trench in the region of the large shallow slip show the presence of horst and graben structure on the subducting plate^23–25^. The 1896 Sanriku tsunami earthquake, close to the Tohoku-Oki earthquake, occurred in the horst and graben structure^26^.

If “asperity” corresponds to areas of relatively high frictional strength^27^, the shear and normal stresses in Fig. 3a,b suggest that the ME of the pre-rupture fault of the Tohoku-Oki earthquake may host a large asperity composed of two asperities, mainly induced by relatively high frictional coefficient and relatively low pore pressure with respect to their surroundings and the asperities favor the horst with higher frictional coefficient (Fig. 2i) and normal stress (Fig. 3b), in which the stress drops and pore-fluid pressure reduction (Fig. 2k) are significant. We may suggest that the horst and graben structure provide a geological environment for the interseismic stress accumulation^26^. In the geological environment the horst contacts the overriding plate tightly to create the asperity with more microcracks and lower pore-fluid pressure, and the graben has less microcracks to support higher pore-fluid pressure close to lithostatic pressure in impending earthquake phase. The former leads to its higher shear stress strength to set up the asperity and the latter leads to its lower shear stress strength to produce huge displacement easily at the trench. It is the horst and graben structure that breed the Tohoku-Oki megathrust earthquake.

## Methods

### Methods to recover mechanical environment of earthquake fault

Whether an earthquake will occur to depend on the ME of the fault. However, little is known about it. If the fault geometry is basically determined, the stress change caused by this earthquake is mainly affected by the ME. This means that the stress change carries the information of the ME. Here, we propose a method to recover a possible integrate ME in the rupture zone of the Tohoku-Oki earthquake as follows.

Taking the inverted stress change (vector)^18^ on the fault as loading of a boundary value problem of the stress model (Fig. S1), we solve a coseismic displacement field by finite element method, and then using this displacement field and displacement–strain relationship and constitutive equation, we calculate the stress change (a tensor) at the fault. This stress change is supposed to represent a stress change close to and in the fault zone, where some foreshocks and aftershocks occurred. To use the stress change and some constraints allows us to predict the ME of the fault before and after the mainshock.

In this paper, we aim to search such a possible integrate ME in the fault rupture zone of the Tohoku-Oki earthquake, that can produce the stress change caused by the earthquake. Here, the integrated ME only includes principal stress state (magnitude and direction), fault friction strength and pore-fluid pressure.

As the Tohoku-Oki earthquake is a pure thrusting event, we use the azimuth and dip angle of maximum principal stress, $\lambda$ and $\theta$, respectively, to define the directions of the maximum, intermediate and minimum principal effective stresses^16^, *S*_1_, *S*_2_, and *S*_3_. In consideration of the effect of pore-fluid pressure on stress state^14^, the stress change caused by the earthquake is taken as the sum of the total effective stresses change (compressive stress as positive) and the pore-fluid pressure change before and after the earthquake,

1 $$ \Delta \sigma_{ij} = s_{ij}^{a} (S_{1}^{a} ,S_{2}^{a} ,S_{3}^{a} ,\lambda_{{}}^{a} ,\theta_{{}}^{a} ) - s_{ij}^{b} (S_{1}^{b} ,S_{2}^{b} ,S_{3}^{b} ,\lambda_{{}}^{b} ,\theta_{{}}^{b} ) + \Delta p\delta_{ij} ,\quad (i,j = 1,\,2,\,3) $$

where $\Delta \sigma_{ij} { = }\Delta \sigma_{ji} \, (for\;i \ne j)$ is the stress tensor, which is solved by taking the inverted stress change as loading on the fault surface in the earthquake stress model^18^, superscripts ‘*b*’ and ‘*a*’ denote ‘before’ and ‘after’ the earthquake, $s_{ij}$ is the effective stress tensor, which can be expressed in terms of the principle effective stresses, *S*_1_, *S*_2_, and *S*_3_ and their directions **n**, that is

2 $$ s_{ij}^{{}} \, = n_{ik}^{{}} S_{ks}^{{}} n_{sj}^{{}} \, $$

there, same subscripts indicate summation, *i*, *j* = 1, 2, 3, and $$S_{11}^{{}} \equiv S_{1}^{{}}$$,$$S_{22}^{{}} \equiv S_{2}^{{}}$$,$$S_{33}^{{}} \equiv S_{3}^{{}}$$,$$S_{ij}^{{}} = S_{ji}^{{}} = 0$$ for $$i \ne j$$ and $$n_{11}^{{}} = \cos \theta \cos \lambda$$,$$n_{12}^{{}} = \cos \theta \sin \lambda$$,$$n_{13}^{{}} = - \sin \theta ,$$$$n_{21}^{{}} = - \sin \lambda$$,$$n_{22}^{{}} = \cos \lambda$$,$$n_{23}^{{}} = 0$$,$$n_{31}^{{}} = \sin \theta \cos \lambda$$,$$n_{32}^{{}} = \sin \theta \sin \lambda$$,$$n_{33}^{{}} = \cos \theta$$, $$\Delta p$$ is the pore-fluid pressure change in the fault rupture zone, and $$\delta_{ij}$$, the Kronecker delta. There are 11 unknowns in Eq. (1),$$S_{1}^{b} ,S_{2}^{b} ,S_{3}^{b} ,S_{1}^{a} ,S_{2}^{a} ,S_{3}^{a} ,\lambda^{b} ,\theta^{b} ,\lambda^{a} ,\theta^{a} ,\Delta p$$, but there are only 6 equations, so we need to add other equations. If it is assumed that rock rupture obeys the Coulomb failure criteria^16^ and that the fault is in an undrained state during earthquake rupture^17^, that may allow us to recover the ME of the earthquake to get a definite solution.

The fault geometry is irregular (Fig. S1), which leads to different normal direction of the fault in the stress model. In consideration of the ME to be recovered is defined in the ruptured area, which is composed of 49 sub-faults with constant dip angle (11°)^18^, each size is 25 × 25 km^2^. The maximum curvature of the curved sub-fault is less than 0.005 (nearly a straight line), thus each sub-fault can be approximated as a plane.

Since the foreshocks close to the fault rupture area are almost thrusting events, and their focal mechanism is nearly the same as that of the main earthquake^28^, the occurrence of these earthquakes is supposed to be controlled by the Coulomb failure criterion with a known fault plane^16^,

$$ S_{1}^{b} {\text{[sin(2}}\beta - \phi ) - \sin \phi ] - S_{3}^{b} {\text{[sin(2}}\beta - \phi ) + \sin \phi ] = 2S_{0} \cos \phi $$

where $\phi$ and $S_{0}$ are the angle of internal friction and the cohesion of the fault, $\beta$ is the angle between the fault normal and the maximum principal effective stress. Neglecting the fault cohesion which is much less than the principal stresses, we can obtain

3 $$ S_{1}^{b} {/}S_{3}^{b} { = (}1 + \mu_{b} \tan \beta )/(1 - \mu_{b} \cot \beta ) $$

where $\mu_{b} = \tan \phi_{b}$, the coefficient of internal friction.

Considering the aftershocks close to the rupture zone are basically normal fault earthquakes^28^ with different strike and dip angle, we suppose that the fracture planes of these earthquakes were controlled by the Coulomb failure criterion with an unknown slip plane,

4 $$ S_{1}^{a} {/}S_{3}^{a} = \left( {\sqrt {1 + \mu_{a}^{2} } + \mu_{a} } \right)^{2} $$

where,$\mu_{a} = \tan \phi_{a}$, the coefficient of internal frictional. This criterion can be derived by $\beta = \pi /4 + \phi_{a} /2$ in Eq. (3).

In coseismic stage, the pore-fluid pressure change $\Delta p$ is supposed in undrained condition^17^ and

5 $$ \Delta p = B(\Delta \sigma_{11} + \Delta \sigma_{22} + \Delta \sigma_{33} )/3 $$

where $\Delta \sigma_{11}$, $\Delta \sigma_{22}$ and $\Delta \sigma_{33}$ are the total normal stress changes, respectively, *B* is the Skempton coefficient, a parameter reflecting the capacity of pore-fluid pressure change under one unit change of mean normal stresses.

As the value ranges of the direction of pre-seismic maximum principal stress, $\lambda_{{}}^{b}$,$\theta_{{}}^{b}$, are known better from observation^13^, thus if the coefficients of frictions $\mu_{b}$ and $\mu_{a}$ are given, Eqs. (1–5) allow us to uniquely solve the ME in the fault rupture zone, that is $S_{1}^{b} ,S_{2}^{b} ,S_{3}^{b} ,S_{1}^{a} ,S_{2}^{a} ,S_{3}^{a} ,\lambda^{a} ,\theta^{a} ,B$. The pore-fluid pressure change $\Delta p$ can be calculated by Eq. (5) using the Skempton coefficient *B* and normal stress changes). Considering the accepted ranges of $\lambda_{{}}^{b}$,$\theta_{{}}^{b}$^13^, $\mu_{b}$^29–32^ and $\mu_{a}$^33^, we need to search all solution sets in their possible ranges^30^:

6 $$ \left| {\lambda^{b} } \right| \le 15^{^\circ } ,\quad \left| {\theta^{b} } \right| \le 15^{^\circ } $$

7 $$ 0 \le \mu_{b} \le 0.7 $$

8 $$ \mu_{a} = \left\{ {\begin{array}{*{20}c} {0.85\;\;\left( {for\;\sigma_{n} \le 200{\text{MPa}}} \right)} \\ {0.6\;\;\;\left( {for\;\sigma_{n} > 200{\text{MPa}}} \right)} \\ \end{array} } \right. $$

with the following constraints, $0.5 \le B \le 0.9$^15^, $45^{^\circ } - 0.5\tan^{ - 1} \mu_{a} < \theta^{a} < 135^{^\circ } + 0.5\tan^{ - 1} \mu_{a}$, according to the focal mechanism of the aftershocks, and $S_{1}^{b} > S_{2}^{b} > S_{3}^{b} \ge 0,\;S_{1}^{a} > S_{2}^{a} > S_{3}^{a} \ge 0$.

Equation (1) is a set of nonlinear equations with the direction cosines, we search its solutions using steps of 0.05° both in the ranges of ± 15° for $\lambda^{b}$ and $\theta^{b}$ as well as of ± 90° for the unknown.

$\lambda^{a}$ and $\theta^{a}$, respectively, and 0.01 both for the frictional coefficients and Skempton coefficients.

The solution precision is controlled by $\sqrt {\delta_{11}^{2} + \delta_{22}^{2} + \delta_{33}^{2} + \delta_{23}^{2} + \delta_{31}^{2} + \delta_{12}^{2} } \le e$, where $\delta_{ij} \equiv s_{ij}^{a} - s_{ij}^{b} + \Delta p\delta_{ij} - \Delta \sigma_{ij}$ and *e* is the error threshold tensor, which is set as 0.01 MPa.

Since there exist many possible solutions to meet the constraints above for the given ranges of the $\lambda_{{}}^{b}$,$\theta_{{}}^{b}$, $\mu_{b}$,$\mu_{a}$ and *B* at one sampling point in each sub-fault in the earthquake stress model^18^, we present an average solution to these solutions to represent the ME there. Considering that the solutions related to the principal effective stresses at the sampling point cannot be made a sum directly as they are tensor with different direction, we need to solve an eigenvalue problem of the sum of the stress tensor $s_{ij}$ which is transformed by Eq. 2 using the solutions of the principal effective stresses. The magnitude and direction of the eigenvector are the magnitude and direction of the averaged principal effective stress, which are referred as recovered principal effective stresses.

In consideration of that the accumulated stresses almost all released^8^ and taking the lithostatic pressure after the mainshock as the total vertical stress, we can obtain the magnitude of pre- and post-seismic pore pressures (Table S3) in the rupture zone by using the recovered pore-fluid pressure changes and the vertical effective stresses, which are transformed from the recovered post-seismic principal effective stresses. The total principal stresses, shown in Table S3, in the rupture zone before and after the mainshock are calculated by using the pre- and post-seismic pore-fluid pressures as well as the recovered pre- and post-seismic principal effective stresses.

## Supplementary Information

Supplementary Information.

## Data Availability

All data generated and analyzed during this study are included in its Supplementary Information files.
