# Supplementary material for: Integrated mechanical environment of pre- and post-rupture fault and asperity origin of the 2011 giant Tohoku-Oki earthquake
Source: Sci Rep. 2022 Dec 8;12:21211. doi: 10.1038/s41598-022-25433-6 (PMC9731991; doi:10.1038/s41598-022-25433-6)

**SUPPLEMENTARY INFORMATION**

Integrated mechanical environment of pre- and post-rupture fault and asperity origin of the 2011 giant Tohoku-Oki earthquake

Zhoumin Xie^1,2^, Yongen Cai ^2^*

^1^National Institute of Natural Hazards, MEMC, China.

^2^Institute of theoretical and applied Geophysics, School of earth and space sciences, Peking University, Beijing 100871, China.

*Corresponding author: Yongen Cai ([yongen@pku.edu.cn)](mailto:yongen@pku.edu.cn))

**SUPPLEMENTARY INFORMATION**

Figure. S1 shows the study region in which the integrated mechanical environment (ME) of the Tohoku-Oki earthquake is recovered and earthquake stress model which is used to solve the stress change in the subduction zone.

Table S1 is the solved stress tensor change in the rupture area using the inverted stresses on the fault of the earthquake, that is used to recover the integrated ME.

Table S2 is the recovered integrated ME in the rupture zone 3 hours before and 3 hours after the Tohoku-Oki earthquake, that includes the principal effective stresses, frictional coefficient, pore fluid pressure change and Skempton coefficient.

Table S3 is the predicted pore-fluid pressure and total principal stresses in the rupture area on the fault, that is obtained by using the result in Table S3.

Table S4 is the shear stresses and the effective normal stress in the rupture area on the fault before and after the earthquake respectively, which are transformed by the recovered principal effective stresses in Table S2. We can obtain the changes of the shear and effective normal stresses before and after the earthquake, which are checked by the inverted.

**Fig. S1**.


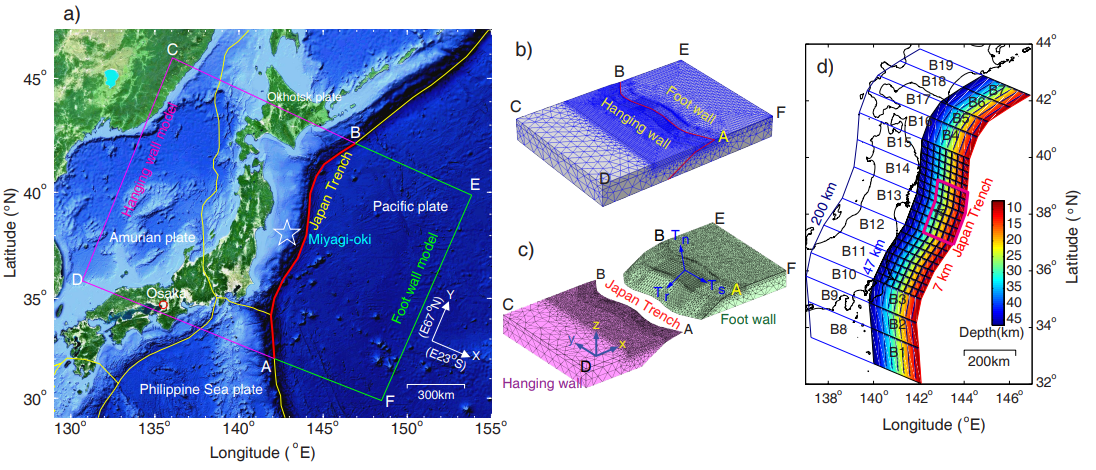
Fig. S1. Study region and earthquake stress model (18). (a) Map of Japan and the Japan Trench from Google Earth (Map data: Google, DigitalGlobe), showing the region of inverse model for fault stress change induced by the Tohoku-Oki earthquake, the fault marked by red line is simplified as an interface across which normal displacement is continuous and the stress vectors on its two sides have the same magnitude but are opposite in direction. (b) Dimensions of the finite element model of the earthquake stress model: 1700 km and 1200 km in the directions normal and parallel to the trench, respectively, and 200 km in the vertical. The fault trace (i.e., the Japan Trench) is marked by a red line. (c) The hanging wall and footwall of the earthquake fault. The three components of the stress on the fault boundary, Tr, Ts and Tn, are only shown on the footwall for clarity. The subscripts r and n denote the directions along and normal to the subduction direction; the subscript s denotes the direction along the fault strike. (d) The sub-faults surrounded by red line is the area in which the mechanical environment of the rupture area of the Tohoku-Oki earthquake is to be recovered. The 19 big sub-faults marked by ‘B’ are far from the mainshock hypocenter, and the 160 small ones are near the hypocenter, the 9th sub-fault along the trench from south to north is numbered 1 in the rupture area.

Table S1.

Solved stress tensors in the rupture area using the inverted stresses.

| ID | Subf | Lon | Lat | Depth | $\Delta\sigma_{xx}$ | $\Delta\sigma_{yy}$ | $\Delta\sigma_{zz}$ | $\Delta\sigma_{xy}$ | $\Delta\sigma_{yz}$ | $\Delta\sigma_{xz}$ |
| --- | --- | --- | --- | --- | --- | --- | --- | --- | --- | --- |
| # | # | (°) | (°) | (km) | (MPa) | | | | | |
| 1 | 9 | 143.52 | 37.08 | 7.5 | -17.98 | -20.98 | -0.16 | 10.19 | -0.86 | 1.19 |
| 2 | 10 | 143.65 | 37.35 | 7.5 | -26.31 | -44.73 | -0.09 | 7.57 | -0.44 | 1.65 |
| 3 | 11 | 143.78 | 37.63 | 7.5 | -24.58 | -40.92 | 0.19 | 1.75 | 0.37 | 1.58 |
| 4 | 12 | 143.91 | 37.90 | 7.5 | -14.65 | -12.58 | -0.04 | -2.24 | 0.48 | 1.05 |
| 5 | 13 | 144.00 | 38.16 | 7.5 | -16.13 | -6.68 | 0.06 | 2.73 | -0.53 | 1.29 |
| 6 | 14 | 144.03 | 38.40 | 7.5 | -26.73 | -32.33 | -1.40 | 6.81 | 0.19 | 0.69 |
| 7 | 15 | 144.07 | 38.64 | 7.5 | -17.67 | -35.70 | -1.62 | 4.67 | 0.91 | 0.70 |
| 8 | 9 | 143.42 | 37.11 | 9.5 | -17.52 | -16.86 | -0.02 | 6.30 | -1.42 | 1.21 |
| 9 | 10 | 143.55 | 37.39 | 9.5 | -27.17 | -35.17 | 0.10 | 5.19 | -0.94 | 1.27 |
| 10 | 11 | 143.68 | 37.66 | 9.5 | -25.74 | -33.76 | 0.22 | 3.39 | 0.46 | 0.91 |
| 11 | 12 | 143.81 | 37.94 | 9.5 | -12.94 | -13.29 | -0.29 | 0.61 | 1.12 | 1.49 |
| 12 | 13 | 143.89 | 38.20 | 9.5 | -14.41 | -11.07 | 0.04 | 1.93 | -0.61 | 1.21 |
| 13 | 14 | 143.93 | 38.44 | 9.5 | -27.93 | -28.99 | -0.59 | 2.43 | -1.28 | 0.41 |
| 14 | 15 | 143.97 | 38.68 | 9.5 | -19.07 | -25.24 | 0.60 | 4.34 | -0.26 | 0.08 |
| 15 | 29 | 143.16 | 37.20 | 14.5 | -25.00 | -13.84 | -0.22 | 0.01 | -3.24 | 2.26 |
| 16 | 30 | 143.29 | 37.47 | 14.5 | -37.34 | -29.26 | -0.03 | 3.41 | -1.47 | 3.67 |
| 17 | 31 | 143.42 | 37.75 | 14.5 | -37.89 | -31.23 | -0.38 | 6.11 | 0.85 | 3.48 |
| 18 | 32 | 143.55 | 38.03 | 14.5 | -28.07 | -13.62 | -0.04 | 3.64 | 1.40 | 1.61 |
| 19 | 33 | 143.63 | 38.28 | 14.5 | -30.18 | -15.80 | -0.18 | 1.74 | -0.65 | 2.26 |
| 20 | 34 | 143.67 | 38.53 | 14.5 | -36.72 | -30.59 | -1.53 | 1.66 | -1.32 | 4.65 |
| 21 | 35 | 143.70 | 38.77 | 14.5 | -22.93 | -16.73 | 0.55 | 4.15 | 0.19 | 2.35 |
| 22 | 49 | 142.90 | 37.29 | 19.5 | -24.94 | -8.88 | 0.64 | -1.94 | -3.21 | 2.13 |
| 23 | 50 | 143.03 | 37.56 | 19.5 | -37.66 | -20.41 | 0.56 | 2.20 | -1.50 | 4.42 |
| 24 | 51 | 143.16 | 37.84 | 19.5 | -38.07 | -20.94 | 0.11 | 5.44 | 1.25 | 4.03 |
| 25 | 52 | 143.29 | 38.11 | 19.5 | -31.13 | -8.17 | 0.57 | 5.17 | 1.78 | 0.68 |
| 26 | 53 | 143.37 | 38.37 | 19.5 | -31.05 | -10.82 | -1.58 | 4.19 | -1.15 | 1.53 |
| 27 | 54 | 143.41 | 38.61 | 19.5 | -29.72 | -15.98 | -0.83 | 2.66 | -1.31 | 5.05 |
| 28 | 55 | 143.44 | 38.85 | 19.5 | -19.81 | -7.80 | 1.09 | 5.10 | -0.39 | 2.98 |
| 29 | 69 | 142.63 | 37.37 | 24.5 | -25.64 | -7.02 | 1.31 | -3.19 | -2.11 | -0.66 |
| 30 | 70 | 142.76 | 37.65 | 24.5 | -37.10 | -14.71 | 1.70 | -0.01 | -0.52 | 1.31 |
| 31 | 71 | 142.89 | 37.93 | 24.5 | -36.48 | -13.64 | 3.00 | 2.98 | 2.37 | 0.66 |
| 32 | 72 | 143.02 | 38.20 | 24.5 | -29.37 | -4.78 | 4.35 | 4.67 | 3.31 | -3.17 |
| 33 | 73 | 143.11 | 38.46 | 24.5 | -28.81 | -4.68 | 3.09 | 4.11 | 1.09 | -2.62 |
| 34 | 74 | 143.14 | 38.70 | 24.5 | -26.09 | -4.01 | 4.40 | 4.17 | -0.27 | 1.24 |
| 35 | 75 | 143.18 | 38.94 | 24.5 | -19.41 | -1.92 | 3.29 | 7.33 | -0.60 | 0.82 |
| 36 | 89 | 142.37 | 37.46 | 29.5 | -27.29 | -5.88 | 1.98 | -5.95 | -1.26 | -3.25 |
| 37 | 90 | 142.50 | 37.74 | 29.5 | -39.89 | -11.37 | 3.08 | -3.62 | -0.30 | -1.93 |
| 38 | 91 | 142.63 | 38.01 | 29.5 | -42.78 | -12.09 | 3.31 | 0.27 | 1.94 | -3.44 |
| 39 | 92 | 142.76 | 38.29 | 29.5 | -39.64 | -9.70 | 2.48 | 3.33 | 3.28 | -7.07 |
| 40 | 93 | 142.85 | 38.55 | 29.5 | -38.34 | -9.32 | 0.91 | 2.94 | 3.01 | -7.46 |
| 41 | 94 | 142.88 | 38.79 | 29.5 | -35.28 | -4.07 | 2.19 | 3.87 | 2.33 | -4.61 |
| 42 | 95 | 142.92 | 39.03 | 29.5 | -24.06 | 0.19 | 2.61 | 7.49 | 1.54 | -2.33 |
| 43 | 89 | 142.27 | 37.50 | 31.5 | -28.93 | -5.83 | 1.03 | -6.43 | -1.51 | -4.67 |
| 44 | 90 | 142.40 | 37.77 | 31.5 | -41.92 | -10.00 | 1.90 | -4.62 | -0.45 | -3.13 |
| 45 | 91 | 142.53 | 38.05 | 31.5 | -46.45 | -11.90 | 1.96 | -1.22 | 1.37 | -5.02 |
| 46 | 92 | 142.66 | 38.32 | 31.5 | -44.25 | -12.22 | 0.29 | 1.64 | 2.41 | -9.78 |
| 47 | 93 | 142.74 | 38.58 | 31.5 | -42.26 | -12.26 | -1.50 | 1.49 | 2.41 | -10.02 |
| 48 | 94 | 142.78 | 38.82 | 31.5 | -37.83 | -4.89 | 0.85 | 2.63 | 2.16 | -6.71 |
| 49 | 95 | 142.81 | 39.07 | 31.5 | -25.83 | -0.55 | 1.13 | 6.65 | 1.53 | -4.32 |

‘ID’ is the order number to calculate stress tensor in the rupture area**; ‘**Subf’, sub-fault number defined in the finite element model; ‘$\Delta\sigma$’ is defined in the coordinate system *oxyz* in Fig.S1c, stress tensor , its first subscript denotes normal direction, the second, force direction.


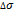


Table S2.

Integrated mechanical environment of the rupture area of the Tohoku-Oki earthquake

| ID | N | S1_b | S2_b | S3_b | Dip_b | Az_b | S1_a | S2_a | S3_a | Dip_a | Az_a | $\Delta p$ | F_a_ | F_b_ | B |
| --- | --- | --- | --- | --- | --- | --- | --- | --- | --- | --- | --- | --- | --- | --- | --- |
|  |  | σ(MPa) | σ(MPa) | σ(MPa) | σ(°) | σ(°) | σ(MPa) | σ(MPa) | σ(MPa) | σ(°) | σ(°) | σ(MPa) | σ | σ | σ |
| 1 | 81 | 29.3 | 23.3 | 19.3 | 4.4 | 127.1 | 28.5 | 25.7 | 6.3 | 92.1 | 170.6 | 9.3 | 0.85 | 0.14 | 0.71 |
|  |  | 2.9 | 3.3 | 3.2 | 1.1 | 9.2 | 2.8 | 2.2 | 0.6 | 0.4 | 6.1 | 1.9 | 0 | 0.06 | 0.14 |
| 2 | 75 | 43.6 | 38.4 | 27.4 | 5.9 | 125.5 | 45.1 | 36.2 | 9.8 | 89.8 | 8.3 | 17.6 | 0.85 | 0.18 | 0.74 |
|  |  | 5.0 | 3.7 | 7.6 | 2.5 | 7.5 | 7.1 | 3.5 | 1.5 | 0.5 | 19.4 | 3.2 | 0 | 0.09 | 0.14 |
| 3 | 174 | 37.9 | 33.1 | 21.1 | 5.6 | 122.4 | 37.2 | 28.9 | 8.0 | 91.2 | 20.3 | 15.8 | 0.85 | 0.26 | 0.72 |
|  |  | 5.7 | 3.5 | 8.3 | 3.1 | 8.4 | 7.8 | 4.7 | 1.7 | 2.4 | 69.1 | 3.0 | 0 | 0.16 | 0.14 |
| 4 | 2141 | 14.9 | 11.2 | 6.9 | 4.4 | 116.9 | 13.3 | 8.4 | 3.2 | 93.8 | 59.1 | 6.4 | 0.85 | 0.33 | 0.71 |
|  |  | 2.5 | 1.5 | 2.6 | 2.2 | 7.9 | 2.7 | 1.8 | 0.7 | 1.3 | 12.8 | 1.3 | 0 | 0.17 | 0.14 |
| 5 | 1118 | 14.8 | 7.5 | 5.3 | 4.5 | 105.6 | 10.6 | 7.1 | 2.6 | 84.9 | 141.5 | 5.2 | 0.85 | 0.44 | 0.68 |
|  |  | 1.3 | 2.2 | 1.7 | 4.0 | 17.2 | 1.8 | 2.3 | 0.5 | 11.1 | 15.3 | 1.1 | 0 | 0.15 | 0.14 |
| 6 | 219 | 32.2 | 26.2 | 15.4 | 2.9 | 125.2 | 28.7 | 22.2 | 6.5 | 91.0 | 0.2 | 14.7 | 0.85 | 0.3 | 0.73 |
|  |  | 5.2 | 3.4 | 6.6 | 1.7 | 8.4 | 6.3 | 3.4 | 1.4 | 2.2 | 19.6 | 2.8 | 0 | 0.17 | 0.14 |
| 7 | 36 | 34.1 | 30.4 | 23.1 | 4.8 | 123.0 | 35.2 | 30.5 | 7.6 | 92.2 | 12.6 | 13.5 | 0.85 | 0.13 | 0.74 |
|  |  | 4.5 | 2.7 | 5.1 | 1.2 | 8.2 | 4.2 | 3.1 | 0.9 | 4.5 | 2.7 | 2.4 | 0 | 0.05 | 0.13 |
| 8 | 468 | 22.2 | 15.3 | 10.2 | 2.1 | 127.6 | 18.6 | 15.2 | 4.4 | 96.2 | 172.8 | 8.3 | 0.85 | 0.29 | 0.72 |
|  |  | 2.7 | 3.1 | 3.5 | 1.7 | 8.1 | 3.2 | 2.3 | 0.8 | 1.5 | 12.4 | 1.6 | 0 | 0.16 | 0.14 |
| 9 | 275 | 34.2 | 27.9 | 16.0 | 3.3 | 124.9 | 31.4 | 22.9 | 7.0 | 88.2 | 8.2 | 15.2 | 0.85 | 0.32 | 0.73 |
|  |  | 5.4 | 3.4 | 6.8 | 1.6 | 9.3 | 6.7 | 4.3 | 1.5 | 3.7 | 38.4 | 2.9 | 0 | 0.17 | 0.14 |
| 10 | 338 | 31.0 | 26.5 | 15.2 | 3.9 | 122.1 | 29.8 | 19.9 | 6.5 | 91.6 | 11.1 | 14.2 | 0.85 | 0.31 | 0.72 |
|  |  | 4.7 | 2.9 | 6.9 | 2.6 | 8.4 | 6.9 | 3.8 | 1.5 | 3.3 | 27.5 | 2.8 | 0 | 0.18 | 0.14 |
| 11 | 5610 | 14.1 | 10.0 | 5.6 | 10.7 | 122.0 | 11.8 | 6.8 | 2.6 | 98.6 | 22.4 | 6.0 | 0.85 | 0.46 | 0.68 |
|  |  | 2.4 | 1.2 | 1.9 | 2.5 | 8.5 | 2.0 | 2.4 | 0.4 | 13.4 | 110.5 | 1.2 | 0 | 0.12 | 0.14 |
| 12 | 6427 | 13.4 | 8.8 | 5.7 | 5.9 | 125.2 | 11.7 | 5.2 | 3.1 | 86.0 | 169.2 | 5.9 | 0.85 | 0.38 | 0.69 |
|  |  | 2.2 | 1.7 | 2.0 | 2.9 | 8.1 | 2.0 | 2.0 | 0.7 | 8.5 | 50.9 | 1.2 | 0 | 0.16 | 0.14 |
| 13 | 200 | 27.5 | 21.0 | 10.5 | 0.0 | 125.0 | 23.6 | 13.0 | 5.5 | 85.9 | 14.2 | 13.5 | 0.85 | 0.36 | 0.71 |
|  |  | 5.9 | 2.7 | 5.3 | 1.5 | 8.1 | 5.0 | 4.5 | 1.2 | 8.0 | 74.2 | 2.8 | 0 | 0.17 | 0.14 |
| 14 | 14 | 25.4 | 20.0 | 11.5 | 0.0 | 129.5 | 22.9 | 17.4 | 5.2 | 89.1 | 9.0 | 10.7 | 0.85 | 0.26 | 0.74 |
|  |  | 4.4 | 2.2 | 4.2 | 0.2 | 8.2 | 3.0 | 2.0 | 0.7 | 1.7 | 44.9 | 1.9 | 0 | 0.12 | 0.13 |
| 15 | 1395 | 26.4 | 12.6 | 9.4 | 7.7 | 115.9 | 19.2 | 10.1 | 6.8 | 75.4 | 31.5 | 8.9 | 0.6 | 0.47 | 0.68 |
|  |  | 3.7 | 1.8 | 2.2 | 4.5 | 5.7 | 2.2 | 3.7 | 0.9 | 22.3 | 106.9 | 1.9 | 0 | 0.11 | 0.14 |
| 16 | 1964 | 37.1 | 25.7 | 16.1 | 8.1 | 124.8 | 32.0 | 14.9 | 11.7 | 86.9 | 177.4 | 15.5 | 0.6 | 0.4 | 0.7 |
|  |  | 5.6 | 4.1 | 5.9 | 3.3 | 7.9 | 6.2 | 5.4 | 2.4 | 7.1 | 68.8 | 3.1 | 0 | 0.15 | 0.14 |
| 17 | 827 | 42.7 | 28.2 | 17.2 | 9.7 | 124.1 | 33.7 | 21.3 | 11.9 | 94.6 | 178.5 | 16.1 | 0.6 | 0.45 | 0.69 |
|  |  | 5.8 | 4.5 | 5.5 | 2.6 | 8.4 | 5.5 | 5.1 | 2.1 | 9.2 | 44.4 | 3.3 | 0 | 0.12 | 0.14 |
| 18 | 685 | 29.0 | 12.7 | 9.8 | 7.5 | 120.4 | 19.7 | 10.9 | 7.3 | 98.2 | 168.5 | 9.4 | 0.6 | 0.5 | 0.67 |
|  |  | 3.1 | 3.1 | 2.5 | 4.2 | 7.0 | 2.6 | 4.0 | 1.3 | 15.7 | 56.1 | 1.9 | 0 | 0.1 | 0.14 |
| 19 | 1134 | 29.4 | 14.7 | 10.7 | 6.2 | 119.9 | 21.3 | 9.7 | 9.4 | 88.3 | 123.2 | 10.6 | 0.6 | 0.46 | 0.69 |
|  |  | 3.8 | 4.3 | 3.2 | 2.6 | 7.5 | 3.2 | 4.8 | 2.8 | 4.8 | 53.6 | 2.2 | 0 | 0.13 | 0.14 |
| 20 | 2347 | 35.6 | 25.8 | 14.6 | 10.3 | 120.6 | 29.5 | 13.8 | 11.0 | 87.1 | 12.0 | 15.7 | 0.6 | 0.44 | 0.69 |
|  |  | 6.3 | 3.5 | 4.7 | 3.2 | 8.0 | 5.1 | 5.6 | 2.2 | 8.0 | 87.0 | 3.3 | 0 | 0.13 | 0.14 |
| 21 | 2066 | 26.2 | 16.0 | 10.2 | 9.9 | 124.5 | 20.3 | 12.6 | 7.3 | 93.3 | 173.8 | 9.0 | 0.6 | 0.46 | 0.69 |
|  |  | 3.2 | 3.2 | 2.9 | 2.4 | 8.5 | 2.9 | 3.3 | 1.2 | 6.6 | 49.7 | 1.8 | 0 | 0.11 | 0.14 |
| 22 | 280 | 28.6 | 10.8 | 9.2 | 10.2 | 108.6 | 18.1 | 11.1 | 6.4 | 73.7 | 53.0 | 6.7 | 0.6 | 0.56 | 0.61 |
|  |  | 3.4 | 2.4 | 1.9 | 4.7 | 8.6 | 2.2 | 4.9 | 0.9 | 19.6 | 68.9 | 1.3 | 0 | 0.06 | 0.11 |
| 23 | 945 | 38.2 | 19.1 | 14.1 | 9.1 | 106.6 | 28.5 | 12.9 | 11.8 | 86.0 | 5.7 | 13.1 | 0.6 | 0.48 | 0.68 |
|  |  | 4.9 | 4.3 | 3.3 | 3.1 | 14.9 | 3.4 | 5.5 | 2.9 | 8.5 | 85.9 | 2.7 | 0 | 0.11 | 0.14 |
| 24 | 605 | 40.9 | 18.8 | 14.3 | 9.7 | 122.5 | 28.6 | 15.6 | 10.6 | 95.7 | 177.9 | 13.2 | 0.6 | 0.51 | 0.67 |
|  |  | 4.7 | 4.2 | 3.0 | 3.1 | 7.8 | 3.2 | 5.8 | 1.8 | 10.1 | 73.3 | 2.6 | 0 | 0.09 | 0.13 |
| 25 | 75 | 33.3 | 13.7 | 10.4 | 7.8 | 105.5 | 20.4 | 16.1 | 8.2 | 79.5 | 146.2 | 8.7 | 0.6 | 0.51 | 0.67 |
|  |  | 2.3 | 3.9 | 3.5 | 7.1 | 15.1 | 3.7 | 4.6 | 1.9 | 15.0 | 26.3 | 1.7 | 0 | 0.1 | 0.13 |
| 26 | 128 | 30.1 | 13.1 | 9.8 | 4.9 | 104.9 | 18.1 | 13.1 | 7.3 | 85.7 | 139.0 | 9.7 | 0.6 | 0.46 | 0.67 |
|  |  | 2.4 | 3.7 | 3.0 | 6.2 | 18.3 | 3.4 | 4.2 | 1.8 | 13.4 | 22.1 | 2.1 | 0 | 0.13 | 0.14 |
| 27 | 1473 | 30.6 | 16.5 | 11.8 | 11.1 | 119.2 | 22.3 | 12.0 | 9.2 | 82.2 | 150.5 | 10.4 | 0.6 | 0.48 | 0.67 |
|  |  | 4.0 | 4.1 | 3.1 | 3.5 | 7.9 | 3.3 | 4.3 | 2.3 | 15.8 | 48.0 | 2.1 | 0 | 0.13 | 0.14 |
| 28 | 656 | 23.6 | 12.1 | 9.3 | 11.2 | 119.2 | 17.0 | 13.7 | 5.8 | 88.5 | 153.2 | 6.0 | 0.6 | 0.47 | 0.68 |
|  |  | 1.9 | 3.4 | 2.7 | 3.0 | 6.5 | 2.7 | 3.6 | 0.9 | 5.6 | 12.2 | 1.2 | 0 | 0.13 | 0.14 |
| 29 | 54 | 28.8 | 13.6 | 10.1 | 9.1 | 113.8 | 20.9 | 16.1 | 7.1 | 108.4 | 83.1 | 7.7 | 0.6 | 0.51 | 0.73 |
|  |  | 1.8 | 3.9 | 2.1 | 3.7 | 6.1 | 2.7 | 4.3 | 0.9 | 36.9 | 11.9 | 1.4 | 0 | 0.09 | 0.14 |
| 30 | 188 | 36.7 | 15.3 | 11.9 | 3.6 | 109.5 | 25.0 | 12.8 | 10.0 | 88.6 | 81.2 | 11.3 | 0.6 | 0.48 | 0.68 |
|  |  | 4.2 | 5.6 | 3.9 | 2.5 | 10.1 | 4.4 | 6.6 | 2.4 | 3.1 | 50.7 | 2.3 | 0 | 0.12 | 0.14 |
| 31 | 152 | 37.6 | 14.8 | 10.9 | 4.6 | 108.1 | 25.3 | 13.5 | 10.2 | 98.2 | 158.9 | 10.9 | 0.6 | 0.51 | 0.69 |
|  |  | 3.8 | 4.1 | 3.7 | 6.9 | 10.6 | 3.9 | 5.0 | 2.3 | 17.4 | 47.2 | 2.1 | 0 | 0.12 | 0.13 |
| 32 | 302 | 33.2 | 5.8 | 3.8 | 9.1 | 100.4 | 15.9 | 8.9 | 7.4 | 107.7 | 43.8 | 6.4 | 0.6 | 0.45 | 0.64 |
|  |  | 2.9 | 4.1 | 3.7 | 16.9 | 23.9 | 3.9 | 5.4 | 2.6 | 17.8 | 60.3 | 1.3 | 0 | 0.14 | 0.13 |
| 33 | 158 | 30.3 | 9.5 | 6.9 | 5.4 | 105.4 | 16.9 | 12.3 | 7.0 | 96.3 | 135.2 | 6.7 | 0.6 | 0.42 | 0.66 |
|  |  | 2.2 | 4.7 | 4.2 | 10.2 | 15.5 | 4.4 | 4.9 | 2.1 | 13.6 | 29.8 | 1.4 | 0 | 0.14 | 0.14 |
| 34 | 118 | 28.5 | 14.4 | 10.9 | 2.6 | 120.9 | 21.3 | 17.1 | 7.4 | 88.0 | 126.9 | 5.9 | 0.6 | 0.41 | 0.68 |
|  |  | 2.2 | 5.8 | 5.1 | 2.2 | 7.6 | 4.9 | 5.4 | 1.7 | 5.5 | 13.7 | 1.2 | 0 | 0.17 | 0.14 |
| 35 | 39 | 23.8 | 8.9 | 7.8 | 3.1 | 96.1 | 15.5 | 14.0 | 5.7 | 96.3 | 140.0 | 4.2 | 0.6 | 0.39 | 0.71 |
|  |  | 1.5 | 3.5 | 3.8 | 7.5 | 33.5 | 3.5 | 3.8 | 1.3 | 12.5 | 6.8 | 0.8 | 0 | 0.17 | 0.13 |
| 36 | 136 | 30.9 | 13.2 | 10.8 | 4.2 | 109.7 | 20.5 | 18.0 | 7.0 | 99.2 | 79.2 | 7.3 | 0.6 | 0.3 | 0.7 |
|  |  | 2.4 | 7.1 | 6.6 | 8.4 | 4.1 | 6.7 | 6.5 | 2.2 | 18.3 | 11.3 | 1.5 | 0 | 0.16 | 0.15 |
| 37 | 126 | 40.6 | 14.8 | 10.6 | 2.6 | -67.0 | 24.6 | 16.8 | 8.9 | 87.6 | 78.1 | 10.9 | 0.6 | 0.44 | 0.68 |
|  |  | 4.0 | 5.7 | 4.0 | 5.2 | 180.4 | 4.2 | 6.5 | 1.7 | 1.2 | 20.3 | 2.2 | 0 | 0.14 | 0.14 |
| 38 | 354 | 42.9 | 10.3 | 6.1 | 6.9 | 109.3 | 21.9 | 12.3 | 8.6 | 98.0 | 58.1 | 11.7 | 0.6 | 0.44 | 0.68 |
|  |  | 4.3 | 2.9 | 2.9 | 13.9 | 8.8 | 2.8 | 5.1 | 1.8 | 8.5 | 58.3 | 2.4 | 0 | 0.14 | 0.14 |
| 39 | 578 | 41.7 | 7.7 | 4.4 | 8.5 | 109.1 | 19.4 | 12.2 | 7.4 | 106.7 | 0.2 | 10.7 | 0.6 | 0.41 | 0.69 |
|  |  | 3.7 | 4.3 | 3.8 | 16.9 | 8.2 | 4.5 | 5.0 | 1.8 | 30.7 | 65.0 | 2.1 | 0 | 0.15 | 0.14 |
| 40 | 496 | 39.3 | 7.8 | 4.5 | 8.5 | 115.0 | 18.2 | 12.2 | 6.7 | 109.7 | 166.8 | 10.8 | 0.6 | 0.39 | 0.69 |
|  |  | 3.3 | 4.9 | 4.3 | 17.1 | 3.2 | 4.9 | 5.2 | 1.8 | 38.3 | 33.3 | 2.2 | 0 | 0.15 | 0.14 |
| 41 | 105 | 37.0 | 7.9 | 5.9 | 10.1 | 102.6 | 16.8 | 11.6 | 8.2 | 100.9 | 137.2 | 7.7 | 0.6 | 0.44 | 0.62 |
|  |  | 2.6 | 7.6 | 6.5 | 16.9 | 17.2 | 7.6 | 8.6 | 3.6 | 20.1 | 51.6 | 1.6 | 0 | 0.13 | 0.13 |
| 42 | 14 | 27.2 | 5.7 | 5.1 | 21.6 | 84.9 | 13.8 | 12.6 | 6.0 | 85.6 | 159.8 | 5.2 | 0.6 | 0.4 | 0.74 |
|  |  | 2.6 | 5.7 | 5.4 | 27.1 | 44.8 | 6.0 | 5.8 | 2.4 | 20.4 | 40.7 | 1.0 | 0 | 0.16 | 0.13 |
| 43 | 72 | 31.6 | 12.5 | 10.0 | 6.7 | 108.3 | 20.3 | 18.4 | 7.1 | 101.2 | 80.9 | 8.4 | 0.6 | 0.25 | 0.75 |
|  |  | 2.4 | 8.1 | 7.0 | 13.2 | 2.1 | 7.2 | 7.2 | 2.4 | 21.9 | 12.5 | 1.6 | 0 | 0.16 | 0.14 |
| 44 | 98 | 42.1 | 14.3 | 10.3 | 4.3 | 113.0 | 23.6 | 18.3 | 8.4 | 86.7 | 81.2 | 11.2 | 0.6 | 0.42 | 0.67 |
|  |  | 3.4 | 6.3 | 5.2 | 8.5 | 4.5 | 5.7 | 6.5 | 2.0 | 1.2 | 17.0 | 2.4 | 0 | 0.16 | 0.15 |
| 45 | 340 | 45.3 | 8.9 | 5.1 | 8.1 | 114.4 | 20.3 | 11.9 | 7.7 | 95.9 | 60.5 | 12.3 | 0.6 | 0.42 | 0.66 |
|  |  | 3.6 | 3.1 | 2.6 | 16.3 | 4.0 | 2.9 | 4.9 | 1.5 | 7.0 | 53.5 | 2.4 | 0 | 0.15 | 0.13 |
| 46 | 229 | 43.6 | 11.4 | 4.5 | 9.1 | 111.4 | 20.0 | 13.7 | 6.9 | 111.7 | 150.9 | 12.5 | 0.6 | 0.36 | 0.67 |
|  |  | 3.2 | 5.3 | 5.4 | 18.1 | 2.7 | 6.4 | 5.4 | 2.1 | 43.1 | 13.5 | 2.6 | 0 | 0.17 | 0.14 |
| 47 | 134 | 40.4 | 11.3 | 4.6 | 9.2 | 111.6 | 19.6 | 13.7 | 6.5 | 115.5 | 144.7 | 13.1 | 0.6 | 0.31 | 0.7 |
|  |  | 3.1 | 4.8 | 5.3 | 18.3 | 3.0 | 5.8 | 5.0 | 1.9 | 51.1 | 9.2 | 2.5 | 0 | 0.16 | 0.13 |
| 48 | 152 | 39.3 | 5.7 | 4.5 | 8.8 | 108.9 | 15.3 | 10.8 | 6.3 | 109.1 | 157.6 | 8.2 | 0.6 | 0.45 | 0.59 |
|  |  | 2.1 | 5.3 | 4.4 | 16.7 | 6.5 | 5.7 | 6.1 | 2.3 | 33.8 | 57.5 | 1.8 | 0 | 0.13 | 0.13 |
| 49 | 16 | 30.0 | 11.0 | 10.4 | 0.8 | 123.6 | 18.7 | 17.8 | 6.2 | 111.5 | 93.0 | 5.5 | 0.6 | 0.39 | 0.66 |
|  |  | 2.5 | 6.5 | 6.3 | 4.2 | 2.2 | 6.4 | 6.4 | 2.0 | 43.9 | 41.2 | 1.2 | 0 | 0.16 | 0.14 |
|  |  |  |  |  |  |  |  |  |  |  |  |  |  |  |  |

‘ ID’ is the same as that in Table S1; ‘N’, the number of searched solutions in a sub-fault$;$ S1,S2 and S3 denote recovered the maximum, intermediate, minimum principal effective stresses, respectively; ‘b’ and ‘a’ denote and before and after the mainshock, ‘Dip’ and ‘Az’, the dip angle and azimuth of the maximum principal stress, the azimuth starts from north and increases clockwise (= 113°- the azimuth defined in the earthquake stress model) ; ‘$\Delta p$’ and ‘B’, pore-fluid pressure change and Skempton coefficient; ‘Fa’ and ‘Fb’ are the internal friction coefficients of the foreshock and aftershock faults, respectively. The boldface in the table is the standard deviation of the number above it.

Table S3. Predicted pore-fluid pressure and total principal stresses in the rupture area.

| ID | Depth | *p_b_* | *σ_*1b | σ_2b | *σ_*3b | *p_a_* | *σ_*1a | *σ_*2a | *σ_*3a | *P_r_* | *R_b_* | *R_a_* | *eμ_b_* | *eμ_a_* | *τ_g_* |
| --- | --- | --- | --- | --- | --- | --- | --- | --- | --- | --- | --- | --- | --- | --- | --- |
| # | (km) | (MPa) | (MPa) | (MPa) | (MPa) | (MPa) | (MPa) | (MPa) | (MPa) | (MPa) |  |  |  |  | (MPa) |
| 1 | 7.5 | 74.5 | 103.9 | 97.9 | 93.9 | 65.3 | 93.9 | 91.1 | 71.7 | 93.8 | 0.794 | 0.696 | 0.028 | 0.041 | 3.6 |
| 2 | 7.5 | 66.4 | 110.0 | 104.8 | 93.8 | 48.7 | 93.8 | 84.9 | 58.5 | 93.8 | 0.708 | 0.520 | 0.053 | 0.087 | 5.1 |
| 3 | 7.5 | 72.7 | 110.3 | 105.5 | 93.5 | 56.6 | 93.8 | 85.5 | 64.6 | 93.8 | 0.775 | 0.603 | 0.059 | 0.103 | 4.0 |
| 4 | 7.5 | 86.9 | 101.8 | 98.1 | 93.8 | 80.5 | 93.8 | 88.9 | 83.7 | 93.8 | 0.927 | 0.858 | 0.024 | 0.047 | 1.3 |
| 5 | 7.5 | 88.5 | 103.2 | 95.9 | 93.7 | 83.2 | 93.9 | 90.4 | 85.9 | 93.8 | 0.944 | 0.887 | 0.026 | 0.050 | 1.0 |
| 6 | 7.5 | 78.4 | 112.0 | 106.0 | 95.2 | 65.1 | 93.8 | 87.3 | 71.6 | 93.8 | 0.836 | 0.694 | 0.045 | 0.093 | 2.6 |
| 7 | 7.5 | 70.7 | 106.3 | 102.6 | 95.3 | 58.7 | 93.9 | 89.2 | 66.3 | 93.8 | 0.754 | 0.625 | 0.030 | 0.049 | 4.0 |
| 8 | 9.5 | 134.6 | 156.8 | 149.9 | 144.8 | 126.1 | 144.9 | 141.5 | 130.7 | 144.8 | 0.930 | 0.871 | 0.020 | 0.037 | 1.9 |
| 9 | 9.5 | 128.7 | 162.8 | 156.5 | 144.6 | 113.4 | 144.8 | 136.3 | 120.4 | 144.8 | 0.889 | 0.783 | 0.035 | 0.069 | 3.0 |
| 10 | 9.5 | 129.5 | 160.2 | 155.7 | 144.4 | 115.0 | 144.8 | 134.9 | 121.5 | 144.8 | 0.895 | 0.794 | 0.034 | 0.064 | 2.9 |
| 11 | 9.5 | 139.2 | 153.3 | 149.2 | 144.8 | 132.9 | 144.9 | 139.9 | 135.7 | 144.8 | 0.961 | 0.918 | 0.018 | 0.037 | 1.0 |
| 12 | 9.5 | 139.1 | 152.4 | 147.8 | 144.7 | 133.1 | 144.8 | 138.3 | 136.2 | 144.8 | 0.961 | 0.919 | 0.015 | 0.031 | 1.1 |
| 13 | 9.5 | 134.2 | 162.3 | 155.8 | 145.3 | 121.2 | 144.9 | 134.3 | 126.8 | 144.8 | 0.927 | 0.837 | 0.025 | 0.058 | 1.9 |
| 14 | 9.5 | 133.2 | 158.0 | 152.6 | 144.1 | 121.9 | 144.8 | 139.3 | 127.1 | 144.8 | 0.920 | 0.842 | 0.022 | 0.041 | 2.3 |
| 15 | 14.5 | 300.9 | 327.3 | 313.5 | 310.3 | 291.1 | 311.2 | 302.1 | 298.8 | 310.3 | 0.970 | 0.938 | 0.014 | 0.028 | 1.8 |
| 16 | 14.5 | 294.2 | 330.9 | 319.5 | 309.9 | 278.3 | 310.4 | 293.3 | 290.1 | 310.3 | 0.948 | 0.897 | 0.021 | 0.041 | 3.1 |
| 17 | 14.5 | 293.2 | 335.5 | 321.0 | 310.0 | 276.6 | 310.4 | 298 | 288.6 | 310.3 | 0.945 | 0.891 | 0.025 | 0.049 | 3.3 |
| 18 | 14.5 | 300.5 | 329.3 | 313.0 | 310.1 | 290.6 | 310.6 | 301.8 | 298.2 | 310.3 | 0.968 | 0.936 | 0.016 | 0.032 | 1.9 |
| 19 | 14.5 | 299.7 | 329.0 | 314.3 | 310.3 | 289.1 | 310.4 | 298.8 | 298.5 | 310.3 | 0.966 | 0.931 | 0.016 | 0.031 | 2.0 |
| 20 | 14.5 | 295.8 | 332.2 | 322.4 | 311.2 | 280.8 | 310.4 | 294.7 | 291.9 | 310.3 | 0.953 | 0.905 | 0.019 | 0.042 | 2.6 |
| 21 | 14.5 | 300.1 | 325.3 | 315.1 | 309.3 | 290.0 | 310.4 | 302.7 | 297.4 | 310.3 | 0.967 | 0.935 | 0.017 | 0.030 | 2.1 |
| 22 | 19.5 | 468.2 | 495.7 | 477.9 | 476.3 | 459.3 | 478.5 | 471.5 | 466.8 | 477.4 | 0.981 | 0.962 | 0.012 | 0.020 | 1.9 |
| 23 | 19.5 | 463.3 | 500.3 | 481.2 | 476.2 | 448.9 | 477.5 | 461.9 | 460.8 | 477.4 | 0.970 | 0.940 | 0.015 | 0.028 | 2.9 |
| 24 | 19.5 | 463.1 | 503.1 | 481.0 | 476.5 | 448.8 | 477.6 | 464.6 | 459.6 | 477.4 | 0.970 | 0.940 | 0.016 | 0.031 | 2.8 |
| 25 | 19.5 | 467.0 | 499.5 | 479.9 | 476.6 | 457.0 | 477.9 | 473.6 | 465.7 | 477.4 | 0.978 | 0.957 | 0.012 | 0.021 | 2.1 |
| 26 | 19.5 | 467.6 | 499.2 | 482.2 | 478.9 | 459.3 | 477.5 | 472.5 | 466.7 | 477.4 | 0.979 | 0.962 | 0.008 | 0.017 | 1.6 |
| 27 | 19.5 | 465.6 | 496.4 | 482.3 | 477.6 | 455.1 | 477.7 | 467.4 | 464.6 | 477.4 | 0.975 | 0.953 | 0.012 | 0.022 | 2.2 |
| 28 | 19.5 | 468.0 | 490.0 | 478.5 | 475.7 | 460.4 | 477.4 | 474.1 | 466.2 | 477.4 | 0.980 | 0.964 | 0.011 | 0.017 | 2.1 |
| 29 | 24.5 | 634.3 | 661.4 | 646.2 | 642.7 | 623.5 | 645.8 | 641 | 632 | 644.4 | 0.984 | 0.968 | 0.009 | 0.015 | 2.2 |
| 30 | 24.5 | 632.5 | 667.4 | 646.0 | 642.6 | 619.4 | 644.4 | 632.2 | 629.4 | 644.4 | 0.982 | 0.961 | 0.010 | 0.019 | 2.6 |
| 31 | 24.5 | 633.6 | 668.1 | 645.3 | 641.4 | 619.2 | 644.9 | 633.1 | 629.8 | 644.4 | 0.983 | 0.961 | 0.011 | 0.019 | 2.6 |
| 32 | 24.5 | 640.6 | 668.9 | 641.5 | 639.5 | 628.6 | 645.3 | 638.3 | 636.8 | 644.4 | 0.994 | 0.975 | 0.006 | 0.010 | 1.6 |
| 33 | 24.5 | 637.6 | 664.6 | 643.8 | 641.2 | 627.5 | 644.6 | 640 | 634.7 | 644.4 | 0.989 | 0.974 | 0.007 | 0.011 | 1.9 |
| 34 | 24.5 | 633.5 | 657.6 | 643.5 | 640.0 | 623.2 | 644.5 | 640.3 | 630.6 | 644.4 | 0.983 | 0.967 | 0.010 | 0.013 | 2.9 |
| 35 | 24.5 | 636.6 | 657.1 | 642.2 | 641.1 | 629.0 | 644.6 | 643.1 | 634.8 | 644.4 | 0.988 | 0.976 | 0.007 | 0.009 | 2.1 |
| 36 | 29.5 | 811.4 | 840.3 | 822.6 | 820.2 | 801.7 | 822.6 | 820.1 | 809.1 | 822.2 | 0.987 | 0.975 | 0.005 | 0.007 | 2.4 |
| 37 | 29.5 | 811.6 | 849.1 | 823.3 | 819.1 | 797.6 | 822.2 | 814.4 | 806.5 | 822.2 | 0.987 | 0.970 | 0.007 | 0.013 | 2.6 |
| 38 | 29.5 | 816.1 | 855.2 | 822.6 | 818.4 | 800.4 | 822.5 | 812.9 | 809.2 | 822.2 | 0.993 | 0.973 | 0.005 | 0.012 | 1.9 |
| 39 | 29.5 | 817.8 | 856.2 | 822.2 | 818.9 | 802.8 | 823.2 | 816 | 811.2 | 822.2 | 0.995 | 0.976 | 0.004 | 0.009 | 1.4 |
| 40 | 29.5 | 817.8 | 855.4 | 823.9 | 820.6 | 804.0 | 823.5 | 817.5 | 812 | 822.2 | 0.995 | 0.978 | 0.003 | 0.008 | 1.1 |
| 41 | 29.5 | 816.3 | 850.7 | 821.6 | 819.6 | 805.4 | 822.8 | 817.6 | 814.2 | 822.2 | 0.993 | 0.980 | 0.005 | 0.009 | 1.6 |
| 42 | 29.5 | 817.2 | 841.5 | 820.0 | 819.4 | 808.4 | 822.9 | 821.7 | 815.1 | 822.2 | 0.994 | 0.983 | 0.004 | 0.006 | 1.5 |
| 43 | 31.5 | 874.9 | 905.2 | 886.1 | 883.6 | 864.7 | 885.5 | 883.6 | 872.3 | 884.9 | 0.989 | 0.977 | 0.003 | 0.006 | 2.1 |
| 44 | 31.5 | 874.7 | 914.7 | 886.9 | 882.9 | 861.3 | 885 | 879.7 | 869.8 | 884.9 | 0.988 | 0.973 | 0.006 | 0.011 | 2.3 |
| 45 | 31.5 | 879.8 | 922.4 | 886.0 | 882.2 | 864.6 | 885.04 | 876.59 | 872.36 | 884.9 | 0.994 | 0.977 | 0.004 | 0.009 | 1.5 |
| 46 | 31.5 | 880.4 | 922.8 | 890.6 | 883.7 | 864.9 | 886.72 | 880.42 | 873.62 | 884.9 | 0.995 | 0.977 | 0.002 | 0.007 | 1.1 |
| 47 | 31.5 | 880.3 | 921.3 | 892.2 | 885.5 | 865.4 | 887.37 | 881.46 | 874.3 | 884.9 | 0.995 | 0.978 | 0.001 | 0.006 | 0.7 |
| 48 | 31.5 | 880.4 | 918.2 | 884.6 | 883.4 | 869.7 | 885.85 | 881.39 | 876.89 | 884.9 | 0.995 | 0.983 | 0.003 | 0.007 | 1.1 |
| 49 | 31.5 | 874.6 | 903.4 | 884.4 | 883.8 | 866.2 | 886.61 | 885.69 | 874.06 | 884.9 | 0.988 | 0.979 | 0.005 | 0.007 | 2.2 |
| mean | 19.5 | 470.0 | 503.0 | 486.4 | 481.4 | 460.0 | 483.0 | 475.8 | 468.0 | 482.5 | 0.950 | 0.907 | 0.016 | 0.029 | 2.2 |

*p_b_*, *R_b_* and *p_a_*, *R_a_* are pore-fluid pressure, pore pressure ratio defined as pore-fluid pressure divided by lithostatic pressure before and after the earthquake, respectively; *σ_ib* and *σ_ia* ( *i*=1,2,3) are maximum, intermediate and minimum principal stresses before and after the earthquake, respectively; *p_r_* is vertical lithostatic pressure; *eμ_b_* and *eμ_a_* are effective frictional coefficient, respectively, before and after the earthquake; *τ_g_* is shear stress on the fault, which is caused by effective gravity; D and *N* are the same as those in Table S2.

Table S4.

Comparison between inversed stresses and recovered stresses in the rupture area on the fault.

| ID | $\tau_{r}^{b}$ | $\tau_{s}^{b}$ | $S_{n}^{b}$ | $\tau_{r}^{a}$ | $\tau_{s}^{a}$ | $S_{n}^{a}$ | $\Delta\tau_{r}$ | $\Delta\tau_{s}$ | $\Delta\sigma_{n}$ | T*_r_* | T*_s_* | T*_n_* |
| --- | --- | --- | --- | --- | --- | --- | --- | --- | --- | --- | --- | --- |
| # | (MPa) | | | | | | | | | | | |
| 1 | 2.5 | -0.4 | 28.0 | -1.9 | 2.3 | 20 | -4.4 | 2.7 | -1.3 |  |  |  |
| 2 | 4.5 | -0.5 | 44.7 | -2.0 | 1.1 | 28.8 | -6.5 | 1.6 | -1.7 |  |  |  |
| 3 | 4.6 | -0.3 | 36.9 | -1.6 | -0.7 | 22.5 | -6.2 | -0.4 | -1.4 |  |  |  |
| 4 | 2.1 | -0.1 | 12.8 | -1.7 | -1.1 | 7.5 | -3.8 | -1.0 | -1.0 |  |  |  |
| 5 | 2.4 | -0.2 | 10.1 | -1.8 | 0.5 | 5.9 | -4.2 | 0.7 | -1.0 |  |  |  |
| 6 | 3.9 | 0.2 | 28.5 | -1.2 | -0.4 | 16.4 | -5.1 | -0.6 | -2.6 |  |  |  |
| 7 | 2.9 | 0.1 | 34.9 | -0.6 | -1.7 | 23.9 | -3.5 | -1.8 | -2.6 |  |  |  |
| 8 | **2.6** | **-0.4** | **18.0** | **-1.8** | **2.1** | **10.8** | **-4.4** | **2.5** | **-1.1** | **-4.3** | **2.5** | **-1.1** |
| 9 | **4.4** | **-0.4** | **30.9** | **-2.0** | **1.3** | **17.2** | **-6.4** | **1.7** | **-1.4** | **-6.4** | **1.5** | **-1.6** |
| 10 | **4.0** | **-0.2** | **29.4** | **-1.9** | **-0.3** | **16.3** | **-5.9** | **-0.1** | **-1.1** | **-6.1** | **-0.2** | **-1.6** |
| 11 | **2.9** | **-0.3** | **11.4** | **-0.9** | **-1.4** | **6.8** | **-3.8** | **-1.1** | **-1.4** | **-3.9** | **-0.9** | **-1.1** |
| 12 | **2.1** | **-0.2** | **11.2** | **-1.7** | **0.2** | **6.3** | **-3.8** | **0.4** | **-0.9** | **-4.0** | **0.4** | **-1.1** |
| 13 | **3.2** | **0.3** | **23.0** | **-2.3** | **0.5** | **11.2** | **-5.5** | **0.2** | **-1.7** | **-5.8** | **0.1** | **-1.4** |
| 14 | **2.5** | **0.2** | **22.6** | **-1.1** | **-0.1** | **12** | **-3.6** | **-0.3** | **-0.1** | **-3.7** | **-1.1** | **-1.0** |
| 15 | **5.2** | **-0.3** | **18.1** | **-1.7** | **2.8** | **11.2** | **-6.9** | **3.1** | **-2.0** | **-6.8** | **3.4** | **-1.9** |
| 16 | **6.4** | **-1.0** | **31.0** | **-4.1** | **0.9** | **18.4** | **-10.5** | **1.9** | **-2.8** | **-10.6** | **2.2** | **-2.8** |
| 17 | **8.4** | **-1.2** | **33.3** | **-2.0** | **-1.0** | **20.4** | **-10.4** | **0.2** | **-3.2** | **-10.3** | **0.0** | **-3.3** |
| 18 | **5.7** | **-0.6** | **19.4** | **-1.1** | **-1.4** | **11.7** | **-6.8** | **-0.8** | **-1.8** | **-7.1** | **-1.0** | **-1.8** |
| 19 | **5.2** | **-0.5** | **20.7** | **-2.6** | **-0.3** | **12.3** | **-7.8** | **0.2** | **-2.1** | **-7.7** | **1.0** | **-2.5** |
| 20 | **7.1** | **-0.3** | **28.5** | **-3.9** | **-0.1** | **17.3** | **-11.0** | **0.2** | **-4.6** | **-10.1** | **0.4** | **-4.3** |
| 21 | **5.1** | **-0.6** | **20.0** | **-1.3** | **-0.9** | **12.2** | **-6.4** | **-0.3** | **-1.2** | **-6.4** | **-0.9** | **-1.5** |
| 22 | **6.4** | **-0.1** | **17.2** | **-0.5** | **2.6** | **11.6** | **-6.9** | **2.7** | **-1.1** | **-7.1** | **2.8** | **-1.2** |
| 23 | **7.8** | **-0.9** | **27.5** | **-3.6** | **0.9** | **17** | **-11.4** | **1.8** | **-2.6** | **-11.5** | **1.9** | **-2.6** |
| 24 | **8.7** | **-1.2** | **27.9** | **-2.3** | **-1.6** | **17.6** | **-11.0** | **-0.4** | **-2.9** | **-11.0** | **-0.4** | **-2.8** |
| 25 | **6.3** | **-0.3** | **20.1** | **-0.4** | **-1.1** | **12.4** | **-6.7** | **-0.8** | **-0.9** | **-6.8** | **-0.9** | **-1.0** |
| 26 | **4.6** | **-0.6** | **17.5** | **-2.2** | **0.9** | **11** | **-6.8** | **1.5** | **-3.1** | **-6.9** | **1.6** | **-2.6** |
| 27 | **6.5** | **-0.6** | **21.1** | **-3.6** | **0.5** | **14.4** | **-10.1** | **1.1** | **-3.7** | **-10.0** | **1.5** | **-3.4** |
| 28 | **4.9** | **-0.4** | **16.7** | **-1.6** | **0.5** | **11.4** | **-6.5** | **0.9** | **-0.7** | **-6.5** | **1.1** | **-0.6** |
| 29 | **6.1** | **-0.1** | **20.6** | **1.5** | **1.3** | **12.4** | **-4.6** | **1.4** | **0.6** | **-5.5** | **1.4** | **0.6** |
| 30 | **6.1** | **-0.4** | **24.5** | **-2.6** | **0.0** | **13.5** | **-8.7** | **0.4** | **-0.3** | **-9.7** | **0.4** | **0.0** |
| 31 | **6.4** | **-0.1** | **24.6** | **-1.8** | **-2.0** | **12.5** | **-8.2** | **-1.9** | **1.2** | **-9.3** | **-1.9** | **1.3** |
| 32 | **1.8** | **-0.1** | **14.6** | **-1.7** | **-2.5** | **4** | **-3.5** | **-2.4** | **4.2** | **-4.5** | **-2.5** | **4.1** |
| 33 | **2.6** | **0.0** | **16.7** | **-0.8** | **-0.7** | **7.2** | **-3.4** | **-0.7** | **2.8** | **-4.3** | **-0.8** | **3.0** |
| 34 | **3.9** | **-0.3** | **20.6** | **-2.9** | **0.4** | **11.9** | **-6.8** | **0.7** | **2.9** | **-7.7** | **0.7** | **2.8** |
| 35 | **2.5** | **-0.7** | **14.8** | **-2.2** | **1.1** | **8.2** | **-4.7** | **1.8** | **2.3** | **-5.2** | **1.8** | **2.1** |
| 36 | **2.5** | **0.1** | **20.5** | **-0.2** | **0.1** | **11.1** | **-2.7** | **0.0** | **2.1** | **-2.6** | **0.1** | **1.7** |
| 37 | **4.5** | **0.0** | **24.3** | **-2.0** | **-0.5** | **11.3** | **-6.5** | **-0.5** | **2.2** | **-6.1** | **-0.5** | **2.0** |
| 38 | **2.8** | **0.0** | **20.8** | **-2.9** | **-2.0** | **6.3** | **-5.7** | **-2.0** | **2.8** | **-5.8** | **-2.1** | **2.9** |
| 39 | **1.8** | **0.0** | **18.7** | **0.3** | **-2.7** | **4.5** | **-1.5** | **-2.7** | **3.5** | **-1.0** | **-3.0** | **3.2** |
| 40 | **1.6** | **0.2** | **17.3** | **1.2** | **-2.8** | **4.6** | **-0.4** | **-3.0** | **2.0** | **0.4** | **-3.4** | **1.8** |
| 41 | **2.3** | **0.1** | **16.1** | **-0.4** | **-1.8** | **6.2** | **-2.7** | **-1.9** | **2.3** | **-2.3** | **-2.1** | **2.4** |
| 42 | **1.7** | **-0.3** | **13.0** | **-0.8** | **-0.4** | **5.3** | **-2.5** | **-0.1** | **2.5** | **-1.9** | **-0.2** | **2.1** |
| 43 | 1.7 | 0.1 | 20.3 | 0.2 | 0.3 | 10.2 | -1.5 | 0.2 | 1.7 |  |  |  |
| 44 | 3.9 | 0.0 | 23.4 | -1.7 | -0.5 | 10.7 | -5.6 | -0.5 | 1.4 |  |  |  |
| 45 | 2.2 | 0.0 | 19.6 | -2.4 | -1.7 | 5.2 | -4.6 | -1.7 | 2.0 |  |  |  |
| 46 | 1.5 | 0.1 | 19.3 | 2.0 | -2.1 | 4.6 | 0.5 | -2.2 | 2.3 |  |  |  |
| 47 | 1.4 | 0.4 | 18.3 | 2.9 | -2.4 | 4.7 | 1.5 | -2.8 | 0.5 |  |  |  |
| 48 | 2.0 | 0.1 | 14.6 | 0.9 | -1.9 | 4.7 | -1.1 | -2.0 | 1.7 |  |  |  |
| 49 | 3.2 | -0.6 | 18.2 | 2.4 | -0.9 | 10.9 | -0.8 | -0.3 | 1.7 |  |  |  |
| mean | **4.5** | **-0.3** | **20.9** | **-1.7** | **-0.2** | **11.4** | **-6.1** | **0.1** | **-0.3** | **-6.2** | **0.1** | **-0.3** |

$\tau_{r}^{b},\tau_{s}^{b},S_{n}^{b}$ and $\tau_{r}^{a},\tau_{s}^{a},S_{n}^{a}$are the shear stresses and the effective normal stress of the sub-fault before and after the earthquake, respectively, which are transformed by the recovered principal effective stresses in Table S2; $\Delta\tau_{r}$, $\Delta\tau_{s}$ and $\Delta\sigma_{n}=S_{n}^{a}-S_{n}^{b}+\Delta p$ are the changes between the corresponding shear and normal stresses before and after earthquake, respectively, where $\Delta p$ is the pore pressure change in Table S2. T*_r_*, T*_s_* and T*_n_* are the inversed stress changes. ID is the same as that in Table S1.


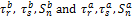

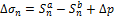

Supplement: Supplementary file 1 — Supplementary Information. [file 41598_2022_25433_MOESM1_ESM.docx]
